# Video-Feedback Interventions Focusing on the Mother–Child Relationship: A Systematic Review of Program Characteristics, Evidence, and Outcomes in Early Childhood

**DOI:** 10.1007/s10567-026-00559-5

**Published:** 2026-03-16

**Authors:** Francesca Favieri, Lucia Lombardi, Cristina Mazza, Alessandra Babore, Cristina Riva Crugnola, Renata Tambelli

**Affiliations:** 1 Department of Psychology, University “G. d’Annunzio” of Chieti-Pescara, Chieti, Italy; 2 Department of Human Neuroscience, Sapienza University of Rome, Rome, Italy; 3 Department of Psychology, University of Milano-Bicocca, Milan, Italy; 4 Department of Dynamic, Clinical and Health Psychology, Sapienza University of Rome, Rome, Italy

**Keywords:** Caregiver, Parenting program, Mother–child interactions, Infant, Video-aided guidance, Mental-health

## Abstract

The quality of parenting is fundamental to the healthy development of children and young people. Evidence indicates that interventions promoting positive and consistent parenting can reduce adverse childhood experiences and improve mental health outcomes. Video-feedback intervention (VFI) is a well-supported technique aimed at strengthening the parent–child relationship, particularly in early childhood. VFI involves recording interactions between a caregiver and a child, followed by the review of selected clips with a trained practitioner. This systematic review examined the effectiveness of stand-alone VFIs, focusing on their characteristics and outcomes for mothers and children aged 0–6 years. A comprehensive search across five major databases (EBSCO, Scopus, WOS, PubMed, and Cochrane Library) yielded 1,451 articles, of which 32 met the inclusion criteria. Results showed that VFI programs are adaptable for mothers of all ages, including adolescents, with interventions ranging from 1 to 15 sessions lasting 20 min to 3 h. They are mainly delivered through home visits, but also in clinical or online settings, and conducted by trained personnel of varying professional levels. Sessions focus on mother–child interactions during activities such as free-play, structured tasks, or daily routines. VFIs improved maternal sensitivity, confidence, and emotional regulation, while enhancing children’s attachment and behavioral outcomes. Effects on parenting stress and maternal representations were mixed. The findings underscore the flexibility and effectiveness of VFIs in supporting mothers from diverse backgrounds and with varying risk factors, all parenting preschool-aged children. The main information of the systematic review protocol was reported in Open Science Framework at the link (https://osf.io/jtg8v/?view_only=2b1809634c3845a2a879003ed03385a8).

## Introduction

The early stages of life constitute a critical developmental window for health professionals, organizations, and intervention programs aimed at promoting well-being. Beginning at conception and encompassing the first 1000 days—marked by rapid physical, cognitive, and socio-emotional growth—this period lays the foundations for multiple human functions. Early childhood development, which some authors extend up to approximately eight years of age (Ardoin & Bowers, 2020), is shaped by early experiences that contribute to self-identity formation and differentiation from caregivers, influencing future developmental trajectories. Research highlights the essential role of caregivers and the significance of daily interactions in shaping long-term outcomes (Knudsen et al., 2014; Shonkoff et al., 2009).

Within this framework, perinatal psychology aims to promote a safe and nurturing environment that supports the well-being of both infants and primary caregivers, whose psychological health directly and indirectly affects all domains of child development. This perspective aligns with WHO guidelines (2020), which emphasize parenting quality as a key determinant of positive developmental outcomes. Positive parenting—encompassing caring, teaching, guiding, and communicating (Seay et al., 2014)—is fundamental for fostering parental sensitivity and responsiveness. Through such interactions, parents serve as secure attachment figures, supporting children’s exploration, emotional regulation, and relationship-building across the lifespan. Early relational experiences thus exert a profound and enduring influence, underscoring the central role of caregiving not only in physical survival but also in shaping psychological and emotional development (Doinita & Maria, 2015; Field, 2010).

Building on these premises, a substantial body of literature has examined how both parents’ psychological well-being and caregiving behaviors influence early childhood development and well-being. These findings have been synthesized in numerous systematic and theoretical reviews (Baldin et al., 2018; Frosch et al., 2021; Lanjekar et al., 2022; Rose et al., 2018). For instance, Panula et al. (2020) found that the psychosocial well-being of both parents during pregnancy predicted children's social competence at age three, highlighting the importance of considering parental mental health within the broader family context. Similarly, a systematic review by Rocha et al. (2020) reported that (i) the quality of mother–infant interactions in the first year of life is significantly associated with children's language, cognitive, motor, and social development at 12 months, and (ii) maternal anxiety may act as a mediating factor negatively affecting developmental outcomes. Furthermore, depressive symptoms in parents—both in the prenatal and postnatal periods—have been shown to negatively impact children's developmental trajectories (La Rosa et al., 2009; Tambelli et al., 2025). Conversely, positive maternal mental health has been associated with more favorable developmental outcomes in infancy, indicating that promoting positive psychological well-being —not merely preventing mental disorders—can offer significant benefits for child development (Phua et al., 2020). Taken together, these findings highlight the crucial importance of interventions that support parental psychological health and enhance the quality of early parent–child interactions, with the goal of optimizing developmental outcomes during infancy.

As highlighted in the recent systematic review by Alves et al. (2024), the development and implementation of evidence-based strategies to promote positive child development and well-being is a global priority, as emphasized by the World Health Organization and UNICEF guidelines (2018). In line with this, numerous support programs have been designed to foster positive parenting and enhance children’s mental health (Alves et al., 2024; Ryan et al., 2017), with their effectiveness also evaluated through randomized controlled trials (see IJzendoorn et al., 2023, for a meta-analysis). Among the various intervention strategies, video-feedback has emerged as one of the most widely adopted and integrated approaches across different programs. Video-feedback intervention (VFI) consists of recording caregiver–child interactions and reviewing selected clips with a trained practitioner. It is a brief, structured approach focused on enhancing caregiver–child exchanges and can be implemented either as part of a multicomponent intervention or as a stand-alone strategy (Juffer et al., 2008). VFIs typically follow three key steps: selecting short naturalistic interaction sequences; identifying moments of relational sensitivity; and discussing these clips with caregivers to foster awareness of the child’s signals, reinforce positive behaviors, and support reflective interpretation of the interaction (Juffer & Bakermans-Kranenburg, 2018; Rutherford et al., 2015; Slade, 2005). The primary goal is to strengthen parental sensitivity and promote secure attachment in early childhood, with growing evidence demonstrating effectiveness across diverse populations (Juffer & Bakermans-Kranenburg, 2018; Poslawsky et al., 2015). Preliminary findings suggest that VFI may positively impact early self-regulatory capacities and the quality of parent–child relational dynamics (Olhaberry et al., 2019). One of the earliest and most comprehensive attempts to evaluate the effectiveness of video-feedback interventions was conducted by Fukkink (2008), who performed a meta-analysis including 29 studies focused on various family support programs that incorporated video-feedback as a core component. The results demonstrated moderate effect sizes across a range of parenting-related outcomes, particularly in improving parental sensitivity, parenting behavior, and parent–child interactions, as well as a medium effect size was reported on improving children behavior. Notably, this meta-analysis included a variety of program types—some of which used video-feedback as part of broader multicomponent interventions—targeting diverse populations beyond infancy. However, Fukkink highlighted the need to further examine the unique contribution of stand-alone video-feedback interventions, particularly during the sensitive and developmentally significant period of early childhood. The importance of conducting a review focused specifically on stand-alone VFI trials lies in understanding their value as interventions that can be delivered in a brief, highly focused, and less costly format. This is especially relevant given that stand-alone VFIs may be as effective as more extensive multicomponent programs, underscoring their potential as accessible and scalable options for supporting early caregiver–child relationships.

More recently, Balldin et al., (2018) conducted a systematic review of video-feedback interventions targeting children, highlighting promising results in improving child behavioral outcomes and caregiver–child relational quality. While their findings further support the utility of video-feedback strategies, they also underscore the heterogeneity of existing programs and the need for more focused evaluations. In particular, the gap in the literature concerning the specific effectiveness of stand-alone video-feedback interventions aimed at improving the mother–child relationship during early development remains, despite empirical research highlight the positive resource in terms of cost-effectiveness. In fact, compared with multicomponent parenting programs, stand-alone VFIs are typically shorts, require limited training for practitioners, can be delivered in multiple and flexible settings (e.g., home-based, clinical, online; Balldin et al., 2018; Provenzi et al., 2020).

Starting from this promising evidence, the present systematic review aims to synthesize, critically examine and eventually update findings on the effectiveness of VFI as a stand-alone technique, in which video-feedback represent the main intervention strategy. The review pursues three main objectives: (1) to identify the target populations in which these interventions have been implemented; (2) to evaluate their effectiveness across various outcome domains (e.g., parenting psychological well-being, child developmental and behavioral outcomes); and (3) to analyze how specific intervention characteristics—such as duration, type of provider, delivery format, and nature of the recorded interactions—may influence intervention outcomes.

## Methods

The Preferred Reporting Items for Systematic Reviews and Meta-Analyses 2020 guidelines (PRISMA; Page et al., 2021) were adopted. The main information of the systematic review protocol was reported in Open Science Framework at the link (https://osf.io/jtg8v/?view_only=2b1809634c3845a2a879003ed03385a8).

### Search Strategy

A systematic electronic search was conducted across five major databases relevant to the topic—EBSCO, Scopus, Web of Science, PubMed, and the Cochrane Library—on 24 November 2024, and was updated on 01 April 2025. The search strategy was applied to all fields and was structured using a combination of MeSH terms and keywords. Full search strings and the number of records retrieved per database are reported in Table 1. Two independent reviewers (F.F. and L.L.) screened titles and abstracts for eligibility according to predefined inclusion and exclusion criteria, using a blinded approach via Rayyan software (Ouzzani et al., 2016), following the automatic removal of duplicates. Discrepancies at this stage were resolved by a third independent reviewer (C.M.). All retained studies were reviewed in full text by all authors. Each full-text article was independently assessed by the three reviewers (F.F., L.L., and C.M.). Disagreements following full-text review were discussed and resolved by consensus. The inter-rater agreement is reported as κ = .89.

**Table 1:** Script and reported record for each database considered

Database	EBSCO* (392 records), Scopus (719 records), Web of Science (101 records), Pubmed (123 records), Cochrane Library (116 records)
1	(*video-feedback* or *video-based*)
2	(*intervention* or *treatment*)
3	(*mother–infant interaction* or *mother–child interaction* or *parent–infant interaction* or *parent–child interaction*)
4	1 AND 2 AND 3

**Note.** Ebsco Database includes: PsychArticle; PsycInfo; Medline; CINAHL Plus; Psychology and Behavioral Sciences Collection

### Inclusion and Exclusion Criteria

Studies were considered eligible for inclusion if they met the following criteria: (i) the participation of mothers; (ii) involvement of preschool-aged children (0–6 years); (iii) use of video-feedback interventions; (iv) focus on the mother–infant interaction; (v) reporting of empirical outcomes; and (vi) publication in English or Italian. The following exclusion criteria were applied: (i) review articles; (ii) commentaries; (iii) theoretical papers; (iv) study protocols; (v) conference proceedings; (vi) qualitative studies; (vii) case reports or case studies; (viii) studies involving fathers; and (ix) interventions combining video-feedback with other components (i.e., dual or multicomponent interventions). No restrictions were placed on the year of publication during the search process.

### Data Extraction and Analysis

Data from the included studies were independently extracted by the three reviewers using a standardized extraction form specifically developed for this review. The extracted information included: study characteristics (authors, year of publication, country, study design), sample details (size, type), intervention features (type, duration, and follow-up), characteristics of the comparison or control group, and outcome measures (types of outcomes, assessment tools, main findings). Indicators of study quality were also recorded. Any discrepancies in the extraction process were resolved through discussion and resolved by consensus.

### Methodological Quality Assessment

The methodological quality of the included studies was assessed using a structured checklist adapted from established critical appraisal tools for quantitative research, including the *Quality Assessment Tool for Quantitative Studies* (Thomas et al., 2004) and the STROBE guidelines for observational studies (von Elm et al., 2007). The checklist comprised 14 items (see supplement 1), grouped into four key methodological domains: Study Design; Methods and procedures; Assessment and Statistical Rigor; Result Interpretation. Each item of the checklist was rated on a 3-point scale (0 = not addressed or clearly inadequate; 1 = partially addressed or insufficiently described; 2 = clearly addressed and appropriate). A percentage score was calculated for each study allowing a categorization based on their quality percentage as follows: high quality (≥ 75%); moderate quality (50–74%); low quality (< 50%).

## Results

**Fig. 1:**
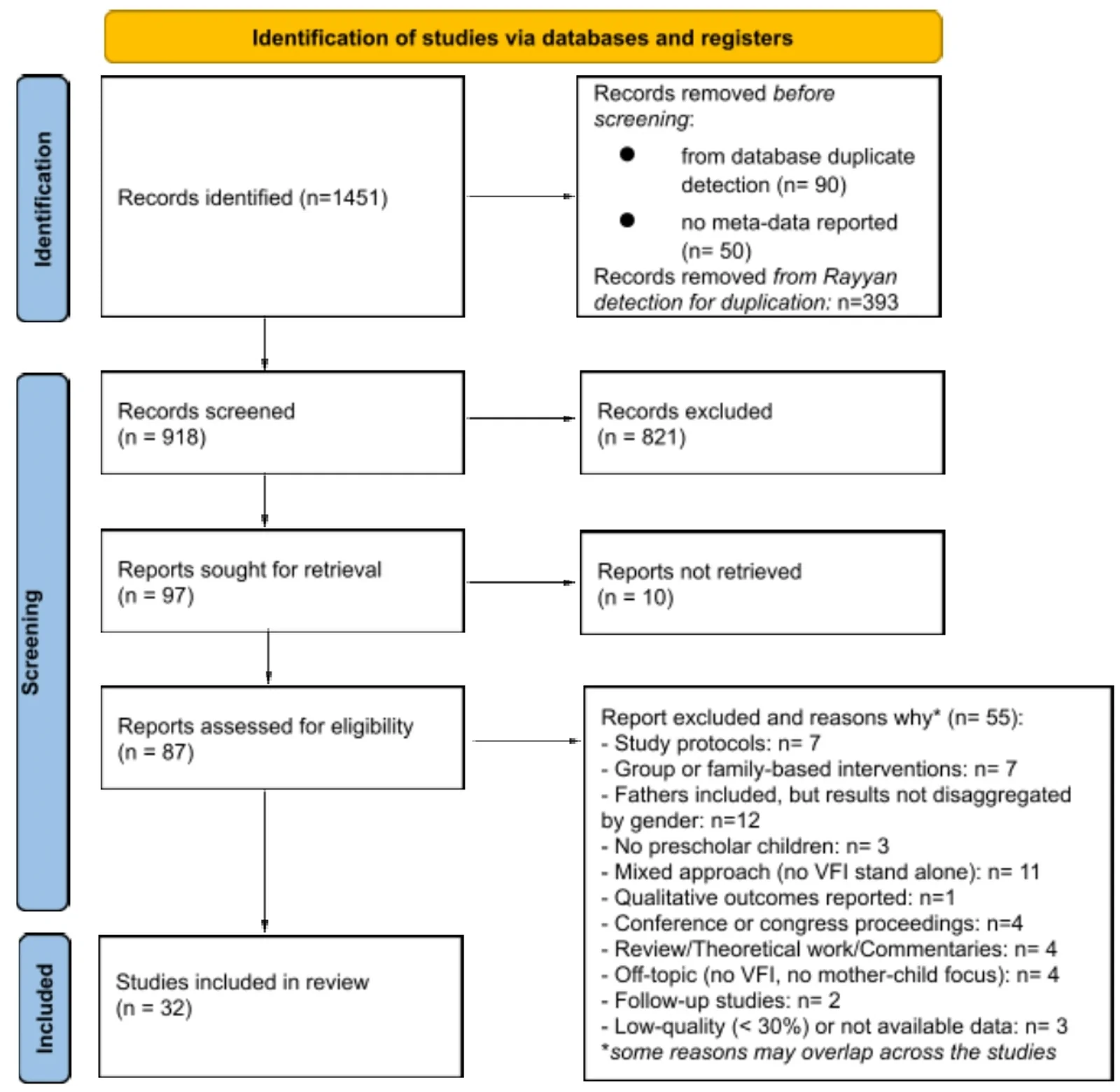
PRISMA (2020) flow diagram outlining screening process. *Note.* This work is licensed under CC BY 4.0. To view a copy of this license, visit https://creativecommons.org/licenses/by/4.0/

The flow diagram in Fig. 1 shows an outline of the systematic search process. One thousand four hundred fifty-one studies were retrieved from databases (*n* = 1451). After excluding Ryyan duplicates (n = 393), nine hundred and eighteen records (n = 918) were screened by title and abstract, and 821 studies were excluded for not meeting the inclusion criteria. Of the studies assessed for eligibility (*n* = 97), ten full texts were not retrieval (*n* = 55). In these cases, the articles were unavailable in any indexed database, and no response was obtained after contacting the authors listed in the records information. Eighty-seven full texts (*n* = 87) were downloaded and analyzed and after the exclusion of fifty-five not eligible studies (see Fig. 1 for exclusion reasons) a final sample of thirty-six records was included in the systematic review (*n* = 32).

### Studies Characteristics

As shown in Fig. 1, thirty-two studies met the inclusion criteria and were included in the synthesis. Their main characteristics are summarized in Table 2. The studies were conducted between 1997 and 2024, across twelve different countries.

**Table 2:** Characteristics of included studies

Author (year)	Country	Aim	Study design	Outcome variable mother	Outcome variable child	Outcome variable interaction	Control Condition	Materials	Sample size	Sample type mother	Sample type child	Results
Alsancak-Akbulut, Sahin-Acar and Sumer (2021)	Turkey	Effectiveness of VIPP-SD in low SES condition	RCT	Sensitivity; Intrusiveness	n/a	n/a	Placebo (“dummy intervention”)	Ainsworth Sensitivity Scale; Cooperation vs. Interference with Baby’s Ongoing Behavior Scale	68 (28 control group; 40 intervention group)	Low to middle SES	n/a	VIPP-SD decreases the level of physical intrusiveness and promotes maternal sensitivity
Alvarenga et al., (2020)	Brazil	Effectiveness of a Short VF intervention (Maternal Sensitivity Program) in low-income parents	RCT	Sensitivity; Intrusiveness	Child Development	n/a	Placebo (“dummy intervention”)	Piccinini et al. Coding (2007); CBDS^7^	44 (22 control group; 22 intervention group)	Low-Income parents	Low-Income children	Intervention increases the ability to interpret meaning of infants’ behavior and communication mothers-babies. Intervention reduces intrusiveness. No effects on infant development
Barnicot et al., (2022)	UK	Feasibility and acceptability of a VIPP-PMH in personality disorders	RCT	Sensitivity; Intrusiveness; Confidence; Stress; Mental health	Behavior problems; Attachment, Emotional functioning and Self-Regulation	n/a	TAU	EAS; BITSEA; CORE-OM; PSOC; PSS; ITSCL	36 (16 control group; 20 intervention group)	Personality Disorders according with DSM-5	n/a	No significant difference and clearly changes emerged in the study’s results
Barnicot et al., (2025)	UK	Effectiveness of VIPP-PMH in personality disorders	Pre-Post intervention (longitudinal)	Sensitivity; Confidence; Intrusiveness; Emotional regulation	Socioemotional difficulties	n/a	n/a	Parent–child interaction	14 mother–child dyads	Personality Disorders according with DSM-5	n/a	Large improvements in parenting confidence and perceptions of the parent-infant relationship. Medium-size improvement in maternal sensitivity
Cassibba et al., (2015)	Italy	Effectiveness of VIPP-R	Pre-Post intervention with control condition	Attachment representations; Sensitivity	Attachment		Placebo (“dummy intervention”)	AAI; EAS; SSP	32 (16 control group; 16 intervention group)	Healthy mothers	Healthy Children	Significant increase of secure attachment and maternal sensitivity
Cimino et al., (2022)	Italy	Effectiveness of VIT for Eating disorders	Pre-Post intervention (longitudinal)	Psychopathological symptoms; emotional and behaviors during feeding	Child Behaviour Problems	Mother–child interaction during feeding	n/a	SVIA; SCL.90; CBCL	342 mother–child dyads	Healthy mothers	Children with eating restriction	Improvement of: maternal emotional state, food refusal in children, dyadic affective state. Reduction of: psychopathological symptoms in mothers (depression, anxiety, OC); internalizing/externalizing prob- lems in children
Cimino and Cerniglia (2024)	Italy	Effectiveness of VIT for Eating disorders	Pre-Post intervention (longitudinal)	Psychopathological symptoms; emotional and behaviors during feeding	Child Behaviour Problems	Mother–child interaction during feeding	n/a	SVIA; SCL.90; CBCL	175 mother–child dyads	Healthy mothers	Children with eating restriction	Improvement of: quality of mother–child interaction Reduction of: maternal psychopathological symptoms in mothers (depression, anxiety); emotional and behavioral symptoms in children
Dubois-Comtois et al., (2017)	Canada	Effectiveness of AVI for neglected children	RCT	Interactive sensitivity; Stress	Neuropsychological development	n/a	TAU	MBQS; PSI; BSID-II	41 (20 control group; 21 intervention group)	Sociodemographical risk factors	Neglected	Improvement in: maternal sensitivity and child mental and psychomotor development No effect: self-reports of stress
Hodes et al., (2017)	Netherlands	Impact of VF based on attachment and coercion theory on parent–child interaction in intellectual disability parents	RCT	Stress; Sensitive discipline; Parental Adaptive functioning	n/a	Harmonious parent–child interaction	TAU	PSS-SF; Semi structured three-bag procedure; Do and Don’t Paradigm	85 (42 control group; 43 intervention group)	Mild Intellectual Disability (MID)	Not specified	Positive effect of VF on parental adaptive functioning in mothers with MID. No effects of VF on sensitive discipline
Juffer et al., (1997)*	Netherlands	Promoting maternal sensitive responsiveness, secure infant-mother attachment relationships, and infant exploratory competence	Pre-Post intervention with control condition*	Sensitivity; Responsiveness	Infant exploratory competence, attachment	n/a	TAU	8-min free-play situation, Strange Situation, Interviews	60 [The first intervention group (N = 30) received a personal book. The second intervention group (N = 30) Book + video feedback group]	Mothers who have adopted a child at 6 months or prior	Adopted children	Positive effect on maternal sensitive responsiveness, infant competence, and presence of secure attachment
Juffer et al., (2005)*	Netherlands	Evaluating the effectiveness of an attachment-based intervention on the prevention of attachment disorganization in adoptive families	Pre-Post intervention with control condition*	Sensitivity; Perceived child temperament	Attachment	n/a	TAU	Dutch Temperament Questionnaire	80 (First intervention group personal book + VF: 50; Second intervention group only book: 30)	Mothers who have adopted a child at 6 months or prior	Adopted children	Enhanced maternal sensitive responsiveness- Children were less likely to be classified as a child with disorganized attachment
Kalinauskiene et al., (2009)	Lithuania and Netherlands	Supporting insensitive mothers with video feedback intervention	RCT	Sensitive responsiveness; Stress; Depressive symptoms; Competence	Attachment	n/a	Placebo (“dummy intervention”)	Waters’ Attachment Q-set, The Daily Hassles scale, Beck Depression Inventory, The Parental Efficacy Questionnaire	54 (26 intervention group, 28 control group)	Insensitivity mothers (diagnosed during free play using Ainsworth’s rating scale)	n/a	Effect of intervention on sensitive responsiveness and attachment security
Kenny et al., (2013)	UK	The study prospectively measured changes in mother-infant interaction following video feedback intervention, during admission to an MBU	RCT	Sensitivity; Responsiveness	Temperament	Mother–child interaction	TAU	CARE-index	140 (22 control group; 49 MBU group; 69 Community ill group)	Mothers with severe mental illness	n/a	Effect of intervention on sensitive responsiveness, infant cooperation. Reduction of passivity
Klein Velderman et al., (2006)	Netherlands	Analyze effects of Attachment-Based Interventions (video feedback) on Maternal Sensitivity and Infant Attachment	RCT	Attachment representations; Sensitivity; Responsiveness;	Temperament, attachment	Mother–child interaction	TAU	AAI, 10 min free play, Strange Situation Procedure, Infant Behavior Questionnaire	81 (27 control group, 28 VIPP, 26 VIPP-R)	Mothers with insecure attachment representation	n/a	Effect of intervention on mother sensitiveness
Klein Velderman et al., (2006)	Netherlands	To evaluate the effects of VIPP and VIPP-R on reducing externalizing behavior problems in preschool-aged children of first-time insecurely attached mothers	RCT	Sensitivity, Perceived social support; Sociability; General health	Child behavior problems: Internalizing, Externalizing, Total Problems, attachment	n/a	TAU	CBCL 2–3, IBQ, AAI, AQS, EAS, baby’s diary, maternal temperament scales (EAS), perceived stress and support questionnaire	81 (27 control group, 28 VIPP, 26 VIPP-R)	Mothers with insecure attachment representation	n/a	VIPP group had significantly fewer children in clinical range for Total and Externalizing problems compared to control; effects not mediated by sensitivity or attachment
Kolijn et al., (2020)	Netherlands	Testing effects of the Video-feedback Intervention to promote Positive Parenting and Sensitive Discipline (VIPP-SD) on mothers’ neural processing of child faces	RCT	Psychopathological symptoms; response to facial stimuli	n/a	n/a	Placebo (“dummy intervention”)	Child Affective Facial Expression database, EEG, Brief Symptom Inventory	66 (44 Control group; 22 Intervention group)	n/a	Typically developing same-sex twins	Promotes efficient, less effortful face processing
Kolijn et al., (2021)	Netherlands	To investigate whether the VIPP-SD improves maternal inhibitory control and leads to better maternal sensitive discipline through improved inhibitory control	RCT	Sensitive discipline, Inhibitory control	Internalizing symptoms	Mother–child interaction	Placebo (“dummy intervention”)	“Don’t touch” task, Ad hoc tasks, he Brief Symptom Inventory, the Adult Temperament Questionnaire	66 mothers (44 control group; 22 intervention group)	Mothers with high levels of maternal inhibitory control	n/a	Maternal inhibitory control did not improve over time in the intervention Group Sensitive discipline slightly decreased in the intervention group compared to control
Kristensen et al., (2017)	Denmark	Investigate whether video feedback promotes a healthy early relationship between infants and vulnerable first-time mothers	Pre-Post intervention with control condition	Confidence, Stress; Mood	infant social emotional behaviors	Dyadic synchrony	TAU	CARE-Index, KPCS, PSS, EPDS, ASQ:SE	278 (209 control group; 69 intervention group)	Mothers living in districts with health visitors certified as Marte Meo therapists	n/a	The intervention group showed improved dyadic synchrony and infant cooperation, higher level of maternal sensitivity and lower levels of parental stress
Lam-Cassettari et al., (2015)	Australia	Test whether the quality of parent– child communication and parental self-esteem would be improved after participation in pilot video-feedback intervention program	RCT	Self-Esteem; Emotional availability; Sensitivity; Structuring, Intrusiveness; Hostility	Child responsiveness, child involvement	n/a	TAU	RSES, EAS	14 (7 control group; 7 intervention group)	Mothers with low levels of emotional disponibility	Congenitally deaf and prelingual children	Video-feedback enhances communication in families with prelingual DHH children and encourages more connected parent–child interaction
Linhares et al., (2022)	Brazil	Examined the efficacy of a personalized remote video feedback parenting program to improve parenting and child behavior outcomes	RCT	Beliefs, values and skills perceived by mothers; maternal practices	Internalizing problems (emotional symptoms and relationship problems) and externalizing problems (conduct problems and hyperactivity)	Mother–child interaction	TAU	PICCOLO, PAFAS, PSOC	92 (42 control group; 50 intervention group)	Mothers from low-to medium-income country	n/a	Direct effect of the intervention to improve the parental sense of competence
Manna and Boursier., (2018)	Italy	Define a brief and video-assisted programme conducted with clinical sensitivity, based on the principles of psycho dynamically oriented observations	Pre-Post intervention (longitudinal)	Representations of family; Stress; Sensitivity; Controlling; Responsiveness	Cooperative, Compulsive, Difficult/threatening, Passive/disarming	Dyadic synchrony	n/a	WFC scale, Parent Development Interview, PSI, CARE-Index,	30 mothers	Healthy mothers	n/a	No significant differences between pre and post intervention in global parenting stress
Negrao et al., (2014)	Portugal	Tested the effectiveness of the VIPP-SD in a sample of poor Portuguese Mothers	RCT	n/a	n/a	Mother–child interaction	Placebo (“dummy intervention”)	EAS, FES	44 (21 control group; 22 intervention group)	Poor Portuguese Mothers (low-income family)	n/a	Dyads in the intervention group showed better functioning from pretest to posttest, whereas dyads in the control group showed no improvement, or even signs of worsening
Pereira et al., (2014)	Portugal	To test the effectiveness of the VIPP-SD program in reducing harsh discipline in mothers at risk for maltreatment	RCT	Harsh discipline (physical, verbal, psychological control)	n/a	Observed discipline in standardized tasks (clean-up and don’t-touch)	Placebo (“dummy intervention”)	Mother–child interaction observations; Parenting Daily Hassles Questionnaire	43 (21 control group; 22 intervention group)	Mothers in severe socioeconomic deprivation, risk for maltreatment	Children aged 1–4 years (mean age 28.44 months)	Harsh discipline significantly decreased only in the intervention group with high parenting stress; no effect in low-stress cases
Piccolo et al., (2024)	USA	To determine the minimal effective dose of the Video Interaction Project (VIP) for improving responsive parenting behaviors in a real-world setting	Pre-Post intervention (longitudinal)	Responsiveness	n/a	Parent–child interactions during play and reading (video-based observation)	n/a	Coach-completed observational checklist, standardized across VIP sessions	183 mother–child dyads (124 completed two follow-ups)	Predominantly low-income, ethnically diverse mothers (71% Spanish-speaking)	n/a	One VIP visit increased responsive parenting by 22%. Findings were independent of child’s age at baseline and time between visits
Riva Crugnola et al., (2016)	Italy	To evaluate the effectiveness of the PRERAYMI an attachment-based intervention programme	Pre-Post intervention with control condition	Mind-mindedness; Sensitivity; Controlling; Responsiveness	Cooperative, Compliant- Compulsive, Difficult, Passive	Quality of the interaction style	TAU	AAI, CECA, Care-Index, Mind-Mindedness Coding Manual	44 mother–child dyads (32 intervention, 12 TAU)	Adolescent and young mothers	n/a	Increased maternal sensitivity and attuned mind-mindedness; decreased controlling behaviors; improved infant cooperativeness in intervention group only
Sandnes et al., (2023)	Norway	To examine if a video-feedback intervention (VIPI) improves maternal representations beyond treatment as usual (TAU) in low-to-moderate risk families	RCT	Maternal representations (WMCI categories and scales)	n/a	n/a	TAU	Working Model of the Child Interview (WMCI), VIPI intervention materials, video recordings	141 (80 intervention, 61 TAU)	Predominantly low-to-moderate risk mothers	n/a	No significant differences in maternal representation improvements between VIPI and TAU; minor improvements noted in total sample over time
Schacht et al., (2017)	UK	To test the feasibility and efficacy of a video-feedback intervention to increase mind-mindedness in mothers hospitalized for severe mental illness (SMI), and to explore its impact on infant–mother attachment	Three-part empirical study (Pre-Post intervention with control condition)	Appropriate and nonattuned mind-related comments	Attachment classification	Infant–mother attachment security	Psychologically well mothers (Study 1 & 2); TAU (Study 3)	Video-recorded mother–infant play sessions; video-feedback intervention; Strange Situation Procedure	Study 1: 99 mother–child dyads (49 mothers psychologically well and 50 hospitalized for SMI), Study 2: 71 mothers (22 completed intervention, 49 TAU), Study 3: 39 (9 intervention, 30 control)	Mothers hospitalized for SMI and psychologically well mother	n/a	Video-feedback intervention reduced nonattuned comments and increased appropriate comments. Infants of intervention mothers more likely to be securely attached compared to standard care
Schechter et al., (2006)	USA	To explore whether a single clinician-assisted videofeedback session (CAVES) can reduce negative maternal attributions toward their young children	Open-trial experimental design with repeated measures (T1, T2, T3)	Maternal Negativity	n/a	n/a	n/a	DTHQ, LEC, BPSAQ, SCID, PCLS, BDI, WMCI, MARS	32 mother–child dyads	Biological mothers with a history of interpersonal violence exposure and PTSD (mean age = 30 years)	n/a	Mothers showed a significant reduction in negative attributions toward their children following a single session of clinician-assisted video feedback. Higher levels of maternal reflective functioning (RF) at baseline were associated with greater reductions in negativity
Stein et al., (2006)	UK	To examine whether videofeedback treatment specifically targeting mother–child interaction would be superior to counseling in improving mother–child interaction, especially mealtime conflict, and infant weight and autonomy	RCT	Eating disorder psychopathology, eating habits and attitudes, depressive symptoms, expectation and perception of treatment	Infant weight and autonomy	Mealtime conflict, maternal facilitation, maternal verbal responses to infant cues, appropriate nonverbal responses to infant cues, and maternal intrusiveness	Supportive counselling	EDEQ, Eating Disorder Examination, EPDS, Mealtime conflict score and Questionnaire on treatment expectation and perceptions	77 (38 VF group, 39 supportive counselling group)	Mothers with bulimia nervosa or bulimic-type EDs	n/a	VF group showed significantly less mealtime conflict and greater infant autonomy than controls. Also showed more maternal facilitation and improved nonverbal responsiveness. No between-group differences in verbal responses, maternal ED or depressive symptoms, or infant weight
Stein et al., (2018)	UK	To assess whether parenting video-feedback therapy (VFT), in addition to CBT, improves child outcomes compared to PMR, in mothers with persistent postnatal depression	RCT	Depressive symptoms	Cognitive and language development, behaviour problems, attachment security, emotion regulation and discrimination, and sustained attention	Maternal responsiveness, emotional scaffolding, mind-mindedness	Active control treatment: Progressive Muscle Relaxation (PMR)	EPDS, SCID-IV-R with CSR, IBQ, BSID-III, CBCL, AQS, ECBQ, Lab-TAB, visual discrimination task observational ratings	144 (72 VFT, 72 PMR)	Mothers with persistent postnatal depression	n/a	No significant differences were found between VFT and PMR groups in maternal interactional variables, depression, or child outcomes
Van Zeijl et al., (2006)	Netherlands	To evaluate the effectiveness of VIPP-SD in improving maternal sensitivity and sensitive discipline in reducing externalizing behaviors in young children	RCT	Sensitivity, discipline (positive/ negative) maternal attitudes (towards sensitivity/towards sensitive discipline)	Child externalizing behavior (Overactive/oppositional/aggressive)	n/a	Six telephone calls discussing general child development (no advice)	Daily hassles; DFPQ; Cantill Ladder; ICQ; CBCL/1.5–5; Maternal attitudes toward sensitivity and sensitive discipline questionnaire; problem-solving tasks for maternal sensitivity; laboratory session with coding procedure for maternal discipline	237 mother–child dyads (120 intervention, 117 control)	Mothers of Dutch origin	Children with elevated externalizing behavior	VIPP-SD improved maternal attitudes and positive discipline; reduced overactive behavior especially in families with high marital discord or daily hassles
Yagmur et al., (2014)	Netherlands	To test the effectiveness of a culturally sensitive adaptation of VIPP-SD (VIPP-TM) in improving sensitivity and discipline strategies in Turkish minority mothers of toddlers with high externalizing problems	RCT	Sensitivity; Intrusiveness discipline strategies	Externalizing problems (CBCL 1½–5)	n/a	Placebo (“dummy intervention”)	CBCL/1½–5; video observations coded with EAS; adapted version of the discipline rating scales used by Verschueren, Dossche, Marcoen, Mahieu, and Bakermans-Kranenburg (2006)	76 families (36 intervention, 40 control)	Second-generation Turkish mothers	Children with, high levels of externalizing problems	Significant increase in maternal sensitivity and nonintrusiveness in intervention group; no significant effect on discipline strategies

VIPP-PMH: video feedback intervention for positive parenting adapted for perinatal mental health; VIPP-SD: Intervention to Promote Positive Parenting-Sensitive Discipline; EAS: Emotional Availability Scale; BITSEA: Brief Infant Toddler Socioemotional Assessment; CORE-OM: Clinical Outcomes in Routine Evaluation Outcome Measure; PSOC: Parenting Sense of Competence scale; PSS: Parental Stress Scale; ITSCL: Infant Toddler Symptom Checklist; CBDS: Child Behavior Development Scale; EPDS: Edinburgh Postnatal Depression Scale; NPI: Neonatal Perception Inventory; PSCS: Parenting Sense of Competence Scale; AAI: Adult Attachment Interview; SSP: Strange Situation Procedure; SVIA: Scala di Valutazione dell’Interazione Alimentare Madre-Bambino; SCL-90: Symptom Checklist-90; CBCL: Child Behavior Checklist; MBQS: Maternal Behavior Q-Set; PSI: Parenting Stress Index; BSID-II: Bayley Scales of Infant Development—II; DAS Dyadic Adjustment Scale; GHS General Health Questionnaire; MBU: In-patient mother and baby units; AAI: Adult Attachment Interview, KPCS: Karitane Parenting Confidence Scale, ASQ:SE-2: Ages & Stages Questionnaires; RSES: Rosenberg Self-Esteem Scale, PICCOLO: Parenting Interactions with Children: Checklist of Observations Linked to Outcomes; PAFAS: Parenting and Family Adjustment Scale; PSOC: Parental Sense of Competence Scale; SDQ: Strengths and Difficulties Questionnaire; FES: Family Environment Scale; EQ-short: Empathy Quotient; BDI: Beck Depression Inventory

*The study included three conditions: a first intervention group in which mothers were asked to write a personal book about their child’s development and their own role as a parent; a second intervention group that involved writing the same personal book, along with participating in three video-feedback sessions; and a third group that received no intervention. For the purposes of this review, we focused only on the two intervention groups, isolating the outcomes related specifically to the video-feedback intervention. This choice determined the final sample size, as the control group was excluded from the synthesis

#### Overview of Study Designs

Approximately one-third were randomized controlled trials (RCTs), while the remaining employed various pre-post designs, including longitudinal pre-post studies (n = 6), and pre-post studies with control groups (n = 7). Among those with control groups, the majority employed placebo conditions (n = 9), treatment as usual (TAU; n = 14), or alternative comparison conditions such as supportive counseling, progressive muscle relaxation (PMR), or telephone-based interventions (Table 3).

**Table 3:** Characteristics of VFI programs

Study	Isolated or associated study	Who perform the intervention	Number of sessions	Time of sessions	Maternal age	Age of the child	Follow up	Place	Video Content
Alsancak-Akbulut, Sahin-Acar and Sumer, (2021)	Isolated	Graduated students after specific training	4 (biweekly thematic session) + 2 booster sessions	n/a	21–42	9 to 30 months	3 months	n/a	10 min unstructured mother–child interaction
Alvarenga et al., (2020)	Isolated	Undergraduate psychology students after training	8	About 45 min	Intervention: 27.18 (5.83) Control: 28.14 (5.81)	3–10 months	n/a	Home visiting	5 min of mother–infant interaction with a set of toys
Barnicot et al., (2022)	Isolated	Experts	6	90 min	16–65	6 to 36 months	8 months	n/a	Everyday activities (mealtime, play with appropriate toys)
Barnicot et al., (2025)	Isolated	Experts	6	90 min	34 (4.3)	2–6 months	5 months	N = 10 at home N = 2 mixed home-based and delivery based N = 2 perinatal mental health clinic	Mother–infant interaction
Cassibba et al., (2015)	Isolated	n/a	5	2 h	33.03 (4.37)	7 to 13 months	n/a	Home visiting	10–15 min of Videotaping at the beginning of each session: Mother–infant interaction
Cimino et al., (2022)	Isolated	Experts	8 (twice a week)	1 h	34.17 (2.31)	24 months	n/a	Online	Videorecording of feeding
Cimino and Cerniglia (2024)	Isolated	Experts	8 (twice a week)	1 h	35.02 (2.45)	24 months	n/a	Online	Videorecording of feeding
Dubois-Comtois et al., (2017)	Isolated	Experts	8 (one a week)	90 min	15–39	1–36 months	n/a	Home-based childcare	15-min videotaped interactive session with toys pro- vided by the intervener
Hodes et al., (2017)	Isolated	Experts	15	n/a	28.06 (6.72)	1–6.5 years	3 months	Home visiting	Exploration versus attachment behavior, the ‘sensitivity chain’ and sharing emotions
Juffer et al., (1997)	Book* + VF	n/a	3	n/a	n/a	6 months	At 12 months (after six months)	Home visiting	Attachment behavior and maternal sensitivity
Juffer et al., (2005)	Book* + VF	n/a	6 (4 home visiting and 2 in laboratory)	n/a	n/a	6 months	At 12 months (after six months)	Mixed (at home and in laboratory)	Attachment behavior
Kalinauskiene et al., (2009)	Isolated	n/a	5	n/a	26.4 (2.94)	7 months	At 12 months	Home visiting	Baby’s contact seeking, playing, exploration and crying behavior and possible reactions to it, understanding the feelings of the baby, sensitive responsiveness to the baby’s signals, and sharing emotions
Kenny et al., (2013)	Isolated	n/a	The number of video feedback sessions in the study reported here ranged from 2 to 9 (mean 2.88, SD 1.32) depending on the length of the mother’s admission to the MBU	n/a	MBU 31.4 (6.3), Community 30.6 (6.6), Control group 28.8 (5.2)	12.4 (12.2) weeks	n/a	Mixed, in the Units and in the Community	Interactions with the child
Klein Velderman et al., (2006)	Isolated	Experts	4	1 h and 30 min	27.8 (3.63)	6.83 (SD 1.03) months	At 11 and 13 months	Home visiting	Interactions with the child
Klein Velderman et al., (2006)	Isolated	Experts	4	1.30 h (VIPP group); 3 h (VIPP-R)	Intervention group *M* = 27.8; (SD = 3.63); Control group *M* = 30; SD 3.63);	7–10 months	When the child is 40 months old	Home visiting	Free play mother–child interaction
Kolijn et al., (2020)	Isolated	n/a	2	n/a	37.95 (4.31)	5.30 years old (SD .60)	4 months	Mixed	Interactions with the child
Kolijn et al., (2021)	Isolated	Experts	6	n/a	37.29 (4.31)	4.66 years old (.60)	n/a	Mixed (home and laboratory	Interactions with the child during specific tasks
Kristensen et al., (2017)	Isolated	Experts	2–6	n/a	30.18 (5.09)	2–6 months	6 months	Home visiting	Interactions with the child
Lam-Cassettari et al., (2015)	Isolated	n/a	6	45 min	n/a	2 years	n/a	Built family room at Research Unit	Typical interaction between the mother and child in the family home
Linhares et al., (2022)	Isolated	n/a	4	90 min	32.42	2–6 years	6 weeks	Online	Free-play and structured-play situations
Manna and Boursier, (2018)	Isolated	Psychologist	4	n/a	31–46 (M = 33; SD = .75)	0–3 years	n/a	Home visiting	Mother–child interaction
Negrao et al., (2014)	Isolated	n/a	6	n/a	29.98 (SD = 6.19; range = 18–46)	1–4 years	n/a	Home visiting	Mother and child interaction
Pereira et al., (2014)	Isolated	4 trained interveners	6 + 2	n/a	18–46	1-to-4-year-old (*M* = 28.4 months, *SD* = 10.4)	n/a	Home visiting	Several mother-interaction tasks, standardize situations (e.g., reading a book, cleaning up)
Piccolo et al., (2024)	Isolated	Non-clinical trained coach	3	n/a	n/a	0–36 months	n/a	Public hospital	Mother–child playing and reading situations
Riva Crugnola et al., (2019)	Isolated	A psychologist and psychomotorist	Average 4.1 videofeedback sessions	n/a	14–21	3–9 months	n/a	Hospital	Free-play situation with suitable toys being available
Sandenes et al., (2023)	Isolated	Trained therapists	6 to 8	Usually 1 h	19–43	1–20 months	6 months	Home visiting	Parent-infant interactions
Schacht et al., (2017)	Isolated	Trained psychologist	1	20 min	23–40	13	In the second year of life	Mother and baby unit	Unstructured play
Schechter et al., (2006)	Isolated	Clinician and research assistant	3	n/a	19–45 (*M* = 30 years)	8–50 months	n/a	Hospital	Free play, separation-reunion, clean up, structured joint attention task
Stein et al., (2006)	Isolated	Trained therapist	13	1 h	18–45	4–6 months	n/a	Home visiting	Mealtime
Stein et al., (2018)	CBT* + VFT or PMR	Trained therapist	11	45 min	18 +	4.5–9 months	In the second year of life	Home visiting or Children’s Service	General interactions and interactions during specific tasks
Van Zeijl et al., (2006)	Isolated	Trained intervener	6	1.5 h	*M* = 30 years	1–3 years	n/a	Home visiting	Mother–child interaction
Yagmur et al., (2015)	Isolated	Trained intervener	6	2.5–3 h	*M* = 29.96 years	18 months and 3 years	n/a	Home visiting	Mother–child interaction

VF: Video-Feedback; MBU: Mother and Baby Unit; VIPP: Video-Feedback Intervention to Promote Positive Parenting; VIPP-R: Video-Feedback Intervention to Promote Positive Parenting with a Representational focus; VFT: Video-feedback therapy; PMR: Progressive muscle relaxation

*The personal book (“*The first year of lif*e”) was delivered to both the intervention groups

**CBT was delivered to both the intervention and control groups

#### Targeted Population

The interventions primarily targeted mothers experiencing various risk conditions, including sociodemographic vulnerability (e.g., low socioeconomic status), histories of interpersonal violence or post-traumatic stress disorder (PTSD), and maternal psychopathology (e.g., severe mental illness, eating disorders, depression, and personality disorders). Some studies also included mothers with intellectual disabilities, insecure attachment states of mind, adolescent mothers, and psychologically healthy mothers. Video-feedback interventions (VFIs) were applied to both low-risk populations and high-risk child populations. These included children with feeding restrictions, histories of neglect, adoption status, externalizing behavioral problems, and those who were congenitally deaf or prelingual.

#### VFIs’ Program Characteristics

VFIs were implemented with mothers of various ages, including adolescents as young as 14. The number of sessions ranged from 1 to 15, with durations spanning 20 min to 3 h. Interventions were most commonly delivered through home-visiting models, though some were conducted online or in institutional settings such as public hospitals or research centers. When specified, VFIs were administered by trained personnel with professional backgrounds ranging from students to licensed psychotherapists. Video content typically focused on mother–child interactions in various contexts, including unstructured free play, structured tasks, or routine activities such as mealtime.

### Evidence and Outcomes

#### Maternal Outcomes

Overall, studies reported improvements in maternal sensitivity and parenting confidence, along with reductions in intrusive behavior. Gains in sensitive discipline and decreases in mental health symptoms (e.g., depression, anxiety, stress) were also observed in several trials, particularly among low-SES samples, mothers with psychiatric conditions, or families facing significant risk. However, some RCTs reported no significant effects on stress or depressive symptoms, indicating variability across populations (e.g., samples involving neglected children or mothers with depression).

Non-randomized studies consistently demonstrated improvements in maternal emotional functioning, parenting stress, and psychopathological symptoms—especially among mothers with eating disorders and adolescent mothers. These studies also documented increases in mind-mindedness and better emotion regulation, suggesting benefits extending beyond sensitivity alone.

#### Child Outcomes

Findings showed improvements in children’s behavioral problems, socioemotional development, and neuropsychological functioning. In adoptive families, interventions were associated with reduced risk of attachment disorganization. Some trials, however, detected no significant effects on infant developmental outcomes despite increases in maternal sensitivity (e.g., studies conducted with low-income Brazilian samples).

Open and pre–post studies likewise reported reductions in internalizing and externalizing symptoms, particularly in interventions targeting feeding disorders. Nonetheless, in many studies, child outcomes were not the primary focus or were not assessed directly.

#### Interaction Outcomes

Interaction quality was a central outcome across nearly all study types.

Research consistently demonstrated improvements in parent–child synchrony, positive discipline, emotional availability, and overall dyadic interaction quality. Several studies also reported increases in children’s cooperative and responsive behavior, as well as more positive and engaged interactions—even in high-risk contexts such as neglect, intellectual disability, and severe maternal mental illness.

Pre–post studies further indicated substantial gains in interaction quality, including reductions in controlling behavior, improvements in feeding interactions, and increases in cooperative behaviors in infants. A minority of studies reported no global improvements, particularly when family stress remained high (e.g., studies involving harsh parenting), highlighting the moderating role of contextual stress.

### Qualitative Assessment of the Risk of Bias

**Fig. 2:**
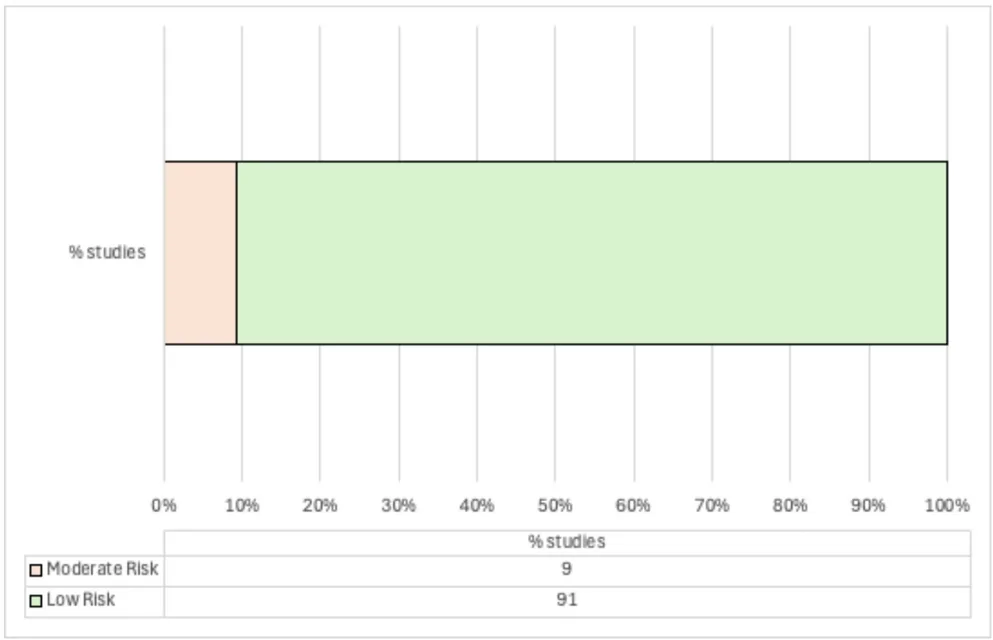
Percentage of risk of bias of the study

**Fig. 3:**
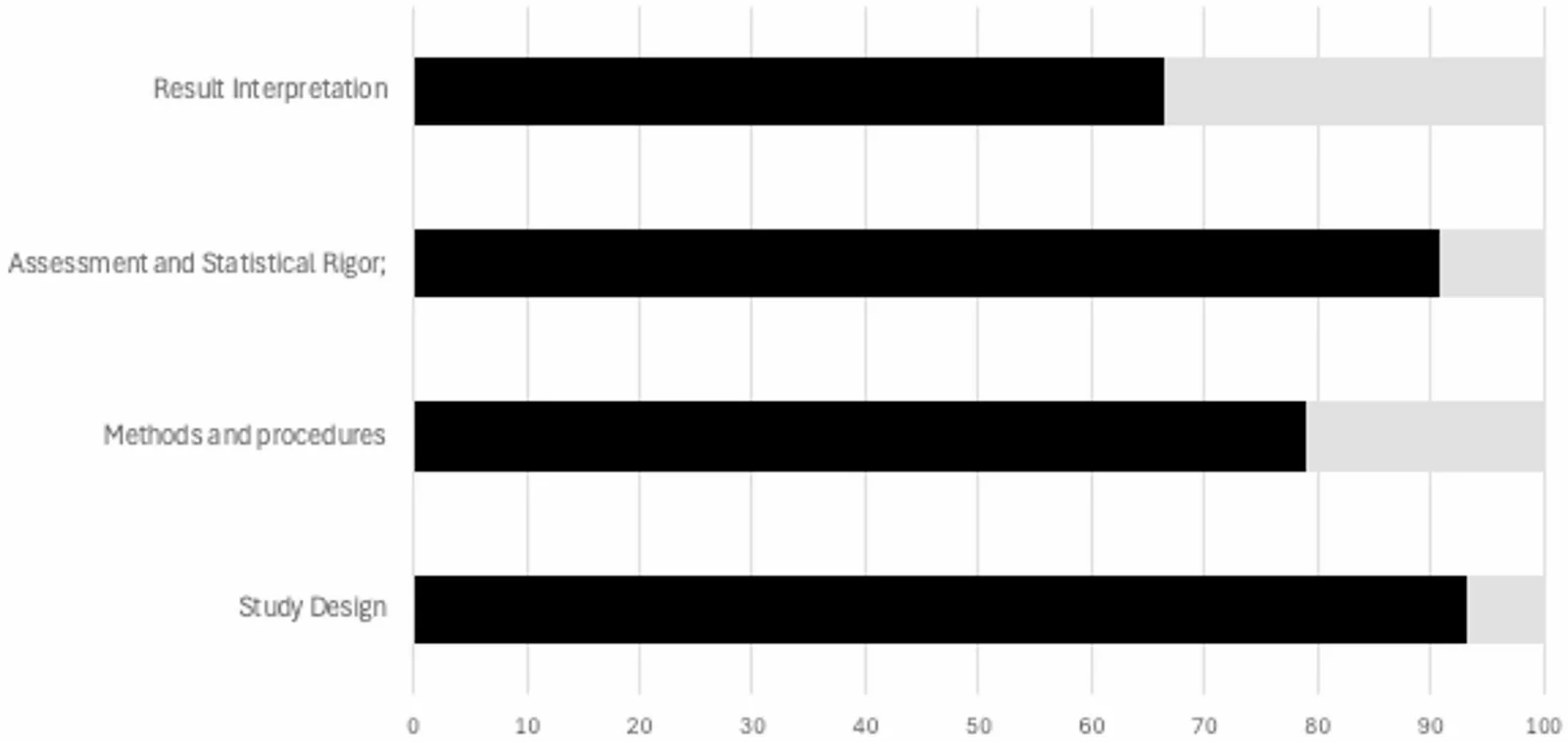
Evaluation of the domains of the quality checklist

The methodological quality of the included studies was generally high. Specifically, 91% of studies (29 out 32) reported high quality, while only 9% (3 out 32) reported moderate risk for bias (see Fig. 2). This indicates a substantial level of methodological rigor across the literature on video-feedback interventions (VFIs), despite heterogeneity in terms of protocol and outcome. Particularly valuable domains were Study Design (1) and Assessment and Statistical Rigor (3), with average ratings exceeding 85%. Most studies provided detailed descriptions of the intervention procedures, participant characteristics, and applied appropriate statistical methods. These strengths reinforce the credibility of the observed intervention effects, especially regarding maternal sensitiveness and interactive behavior. However, the domain Result Interpretation (4) showed moderate quality, with an average score around 70%. Several studies did not explicitly address potential confounding variables or failed to discuss limitations in the interpretation of causal relationships, particularly in non-randomized or small-sample designs (see Fig. 3). This suggests a need for greater attention to internal validity threats and for more nuanced reporting of intervention outcomes. Overall, the quality assessment confirms the methodological soundness of the existing evidence based on VFIs, while also highlighting the importance of strengthening critical reflection and transparency in future studies—especially in domains where interpretative bias may compromise result generalizability.

## Discussion

The current review highlights the broad applicability and promising effectiveness of stand-alone video-feedback intervention across multiple populations of mothers and children, including those with psychosocial vulnerabilities and clinical diagnoses (e.g., Kenny et al., 2013), and socioeconomically disadvantaged backgrounds (e.g., Piccolo et al., 2024). The heterogeneity of provided intervention formats, sample characteristics, and outcome measures shows both the versatility and the nuanced impact of VFIs approaches. The findings of this work are consistent with Fukkink’s meta-analysis (2008), which demonstrated that the duration and dosage of VFIs moderate their effectiveness. In that study, shorter, content-focused interventions proved to be as effective as longer ones when accompanied by structured guidance and feedback. However, variability in session number and follow-up duration across studies complicates cross-study comparisons (Balldin et al. 2018). This qualitative summary supports this evidence, despite the consistency about the positive impact of VFIs, especially on maternal sensitivity. For example, Alsancak-Akbulut et al. (2021) and Alvarenga et al. (2020) found that VIPP-SD and short VF interventions significantly enhanced maternal sensitivity and reduced physical intrusiveness among mothers from low socioeconomic backgrounds. These results are consistent with earlier work by Juffer et al. (2008), who emphasized that maternal sensitivity is a key modifiable factor in the promotion of secure attachment relationships. Also, these results align with findings from Cassibba et al. (2015) and Dubois-Comtois et al. (2017), who reported increased sensitivity and secure attachment patterns following VFIs in healthy and neglected samples. Taken together, these studies support the meta-analytic findings by Van IJzendoorn et al. that, already in 1995, showed that brief, attachment-based interventions are most effective when they focus on enhancing parental sensitivity through feedback and guided observation. Longitudinal studies such as Barnicot et al. (2024) demonstrated medium to large improvements in parenting confidence and sensitivity among mothers with personality disorders, highlighting the feasibility of VFI in more complex clinical contexts. Similarly, Cimino et al. (2022) and Cimino and Cerniglia (2024) showed that repeated video-based interventions significantly improved emotional regulation and interactive patterns in dyads affected by feeding difficulties, with collateral benefits for maternal depression and child emotional and behavioral symptoms.

Hodes et al. (2017) highlighted the importance of VFI in populations with intellectual disabilities, confirming that VFI may function as a compensatory mechanism, enhancing reflective functioning even in contexts of cognitive limitation (Slade, 2005). In line with mentalization-based frameworks (Fonagy & Target, 1997), VFIs provide mothers with an opportunity to observe themselves and their child from a third-person perspective, promoting metacognitive awareness and reflective capacity. This process is particularly relevant in parents that are characterized by a compromission of emotional regulation and mentalization (van der Asdonk et al., 2021). Moreover, some interventions revealed limited or conditional effects. For example, Pereira et al. (2014) noted that VIPP-SD decreased harsh maternal discipline only in cases where mothers reported elevated parenting stress, indicating a moderating role of self-perception in intervention efficacy. Similarly, Kalinauskiene et al. (2009) found improvements in maternal responsiveness but no enhancement in infant attachment security, suggesting that child outcomes may be less susceptible to short-term change. In addition, through some studies emerged the neurobiological basis of VFI effectiveness. Kolijn et al. (2020) underlined that the VIPP-SD influenced mothers’ neural responses to child facial expressions, supporting the issue that VFI may promote more efficient emotional processing and attunement at a cognitive-affective level, aligning with theories of parental reflective functioning (Fonagy & Target, 1997; Rutherford et al., 2015). These findings are supported by other neuroimaging studies, which have shown that parenting interventions can alter neural circuits involved in empathy and emotion regulation (Musser et al., 2012). Such data reinforce the idea that stand alone VFIs work not only at the behavioral level but also by modulating brain-based mechanisms underpinning parental sensitivity via affective networks.

Despite these significant results, there are two studies that reported no significant changes. Manna and Boursier (2018) and Barnicot et al. (2022) reported no significant modification in parenting stress or child development outcomes, despite improvements in maternal perceptions or behavior. These discrepancies may reflect differences in study design, sample size, intervention fidelity, or the measurement of outcomes.

Taken together, the findings suggest that VFI is a flexible and effective approach for improving key dimensions of parenting, such as sensitivity, confidence, and interaction quality, across a wide range of populations and contexts. However, outcomes related to child development and psychopathology appear more variable and may depend on intervention intensity, baseline characteristics, and contextual moderators.

The studies reviewed highlight several important clinical implications for the implementation of video-feedback interventions (VFIs) across diverse caregiving populations. First, VFIs have proven particularly effective in enhancing maternal sensitivity, reducing intrusive behaviors, and fostering positive parent–child interactions—even among high-risk mothers. This evidence suggests that VFIs represent a versatile intervention tool, suitable for integration into various clinical contexts, including perinatal mental health services, pediatric settings, and home-visiting programs.

Second, the consistent improvements observed in parenting confidence and competence following VFI indicate its potential to buffer parental stress and alleviate the emotional burden commonly experienced by vulnerable caregivers. Clinicians may thus consider incorporating VFIs into broader parenting support frameworks to promote caregiver well-being and resilience.

Third, the feasibility and adaptability of VFIs allow for their tailoring to the specific needs of target populations, without altering the core principle of the method. This foundational mechanism facilitates maternal decentering and promotes reflection on parenting behaviors by shifting the focus from self-evaluation as a “good” or “bad” mother to the observation and analysis of specific, concrete interactions. Even when the feedback highlights areas needing improvement, the emphasis remains on behaviors rather than on the mother's identity or worth. This process allows for change without activating self-critical judgment, thus fostering a non-defensive and constructive engagement.

However, clinical application should also be informed by limitations. For instance, effects on child development and attachment security are not always consistent (Kalinauskiene et al., 2009; Barnicot et al., 2022), and certain outcomes may be contingent on moderators like parenting stress (Pereira et al., 2014). This underscores the importance of individualized assessment and the potential need to combine VFI with complementary interventions (e.g., psychotherapy, social support services).

Taking into account the cautions—primarily related to the heterogeneous implementation of the interventions (e.g., duration, setting, target population, and delivery modalities)—this systematic review underscores the potential of video-feedback interventions (VFIs) to inform and strengthen public health strategies, particularly within early intervention services. Their brief and cost-effective format makes them well-suited for integration into universal prevention frameworks, especially in low-resource settings or during high-risk developmental periods such as the perinatal phase, as well as in contexts involving neurodevelopmental disorders (Provenzi et al., 2020). Overall, VFIs emerge as a promising evidence-based approach for enhancing parenting quality and fostering stronger parent–child relationships in both clinical and preventive settings.

## Data Availability

The main information of the systematic review protocol was reported in Open Science Framework at the link https://osf.io/jtg8v/?view_only=2b1809634c3845a2a879003ed03385a8
